# Association of Low-Birth Weight with Malnutrition in Children under Five Years in Bangladesh: Do Mother’s Education, Socio-Economic Status, and Birth Interval Matter?

**DOI:** 10.1371/journal.pone.0157814

**Published:** 2016-06-29

**Authors:** M. Shafiqur Rahman, Tamanna Howlader, Mohammad Shahed Masud, Mohammad Lutfor Rahman

**Affiliations:** Institute of Statistical Research and Training, University of Dhaka, Dhaka-1000, Bangladesh; Swinburne University of Technology, AUSTRALIA

## Abstract

**Background:**

Malnutrition in children under five years remains a significant problem in Bangladesh, despite substantial socio-economic progress and a decade of interventions aimed at improving it. Although several studies have been conducted to identify the important risk factors of malnutrition, none of them assess the role of low birth weight (LBW) despite its high prevalence (36%). This study examines the association between LBW and malnutrition using data from the Bangladesh Demographic and Health Survey (BDHS) 2011 and provides practical guidelines for improving nutritional status of children.

**Methods:**

Malnutrition in children is measured in terms of their height-for-age, weight-for-height, and weight-for-age. Children whose Z-scores for either of these indices are below two standard deviations (–2SD) from median of WHO’s reference population are considered as stunted, wasted or underweight, respectively. The association between malnutrition and LBW was investigated by calculating adjusted risk-ratio (RR), which controls for potential confounders such as child’s age and sex, mother’s education and height, length of preceding-birth-interval, access to food, area of residence, household socio-economic status. Adjusted RR was calculated using both Cochran-Mantel-Haenszel approach and multivariable logistic regression models controlling for confounder.

**Results:**

The prevalence of malnutrition was markedly higher in children with LBW than those with normal birth-weights (stunting: 51% vs 39%; wasting: 25% vs 14% and underweight: 52% vs 33%). While controlling for the known risk factors, children with LBW had significantly increased risk of becoming malnourished compared to their counter part with RR 1.23 (95% CI:1.16–1.30), 1.71 (95% CI:1.53–1.92) and 1.47 (95% CI: 1.38–1.56) for stunting, wasting and underweight, respectively. The observed associations were not modified by factors known to reduce the prevalence of malnutrition, such as higher education of mother, better household socio-economic conditions and longer birth-interval.

**Conclusions:**

Higher education of mother, better household socio-economic conditions and prolonged birth intervals alone are not sufficient in bringing about substantial reductions in prevalence of child malnutrition in Bangladesh. Targeted interventions should be designed to reduce prevalence of LBW in addition to improving mother’s education and other socio-demographic conditions.

## Introduction

Malnutrition is considered as one of the major causes of morbidity and mortality in children under five years of age [1,2]. It has both short-and long-term effects that are detrimental to growth and development of children [3–6]. For instance, malnourished children are physically, emotionally and intellectually less productive than well nourished children and are at an increased risk of suffering from chronic illnesses and disabilities [7–9]. According to 2014 regional estimates, prevalence of malnutrition is highest in South Asian countries and particularly high in India, Pakistan and Bangladesh [10,11]. Although Bangladesh has made rapid progress over the last decade in meeting most of the millenium development goals [12,13], there has been very slow progress in improving the state of child nutrition [14]. The malnutrition rate in Bangladesh remains unacceptably high compared to the developed world [14] despite more than a decade of interventions aimed at improving it. According to the recent national health survey [15,16], the prevalence of stunting (low height for age) reduced from 51% in 2004 to 41% in 2011 while the prevalence of underweight (low weight for age) reduced from 43% in 2004 to 36% in 2011. During the same periods, the prevalence of wasting (low weight for height) remained constant at around 16%, exceeding the WHO emergency threshold level of 15%. These trends suggest that new interventions are required to bring significant improvements in nutritional status of children under five in Bangladesh.

Reducing the burden of malnutrition in children under five years is now one of the major concerns of the government and many international agencies in Bangladesh. Therefore, it is essential to identify the factors associated with malnutrition in children under five so that stakeholders can implement evidence-based policy to improve nutrition status. Identifying these factors and providing practical guidelines to improve nutrition has become one of the main challenges for public health researchers [3]. Studies conducted world wide in the last two decades [17–26] identified maternal illiteracy, household’s low economic status, food insequrity, lack of exclusive breast feeding, administration of pre-lacteals, and deprivation of colostrum as the potential factors associated with child malnutrition. Among the studies conducted in developing countries such as Bangladesh [17–21], low level of mother’s education, poor socio-economic status and short preceding birth intervals were identified as the major risk factors [17,18,21,27]. Bangladesh has demonstrated remarkable gains on each of these fronts over the last decade [15], yet the progress towards reducing the prevalence of child malnutrition in Bangladesh has been disappointingly slow [3,14]. This could be due to the fact that child malnutrition is associated with not just these three factors, but rather, it is the manifestation of the complex interplay of several factors that need to be understood if effective strategies are to be formulated to tackle the problem. In particular, this study examines the association of low birth weight (LBW) with child malnutrition.

LBW is reported in many studies [28–30] as a risk factor for mortality and morbidity in children under five years of age. Although a few studies identify LBW as a correlate of malnutrition [18,21,31] (more references appear in discussion), most of these studies do not treat LBW as the main risk factor of interest. Thus it remains to be seen how and to what extent LBW is associated with malnutrition in children during their early years of life. Given a high prevalence of LBW in Bangladesh [28], this study carries out an indepth analysis to evaluate the importance of LBW in determining malnutrition in relation to other known risk factors using data from the Bangladesh Demographic and Health Survey (BDHS) 2011. It is expected that the findings of this study will help decision makers to design and implement evidence-based policy to improve the nutritional status of children under five in Bangladesh as well as in developing countries having similar nutritional experience.

## Methodology

### Data

Data on child nutrition has been extracted from the database of the Bangladesh Demographic Health Survey (BDHS) 2011 conducted between November 2010 and April 2011. The BDHS is a nationally representative sample survey that has been carried out every two years since 1993 through the collaborative efforts of the National Institute of Population Research and Training (NIPORT),ICF International (USA), and Mitra and Associates under the world-wide demographic and health survey program (DHS). BDHS is a retrospective study based on two stage stratified-cluster sampling design where each of the seven adminstrative divisions was treated as strata. From each stratum the primary sampling units called PSUs (wards in rural and sub-wards in urban areas) were randomly selected at the first stage and households were then randomly selected from each PSU at the second stage. The survey collects health and reproductive history of both men and women of reproductive age. In particular, it collects information on childhood mortality, maternal and child health, nutrition, breast feeding practice and knowledge and attitude regarding HIV/AIDS. Nutrition information has been collected for all living children of age up to 5 years at the time of interview by recording their anthropometric measurements. Children with twin or multiple birth history were excluded from the analysis to avoid correlation in data from multiple births. Therefore, this study is based on 7530 living children of age under five years who have anthropometric data available. Further details may be found in the report on BDHS 2011 [15].

### Eithical considerations

The Eithics committee at NIPORT, Mitra and Associates, and ICF international approved a waiver from eithical approval for this retrospective study. As the de-identified data for this study came from the secondary sources, this study does not require eithical approval.

### Variables

#### Dependent variables

The dependent variable of this study is malnutritonal status (nutritional deficit) in children under-five years that is measured using three different anthropometric indices called stunting (lowheightforage), wasting (low weight for height) and underweight (low weight for age). Each of these indices are expressed as the number of standard deviation (SD) units (Z-score) from the median of the reference population for which the 2006 WHO (World Health Organization) Child Growth Standards were calculated [15,32]. WHO Child Growth Standards are based on an international sample of eithnically, culturally, and genetically diverse healthy children living under optimum conditions that are appropriate for achieving a child’s growth potential [32]. Each of these three indices provides different information about growth and body composition that can be used to assess nutritional status. Height-for-age measures the linear growth of children. A child whose Z-score for height-for-age is more than two standard deviations (-2SD) below the median of the WHO reference population is considered short for his/her age or stunted. Stunting reflects failure to receive adequate nutrition over a long period of time, particularly, during the critical 1000-day period from pregnancy to the child’s second birth day and therefore it is considered as a cummulative effect of chronic malnutrition. Weight-for- height describes acute or current malnutrition that is the consequence of poor dietary intake or frequent occurrence of infectious disease. Children whose Z-scores for weight-for-height are below two standard deviations (-2SD) from the median of the WHO reference population are considered to be too thin for their height or wasted. Weight-for-age is a composite index of the above two indices. Since a child can be underweight for his/her age if he/she is stunted or wasted or both, weight-for-age is an overall indicator of a population’s nutritional health. Children whose Z-scores for weight-for-age are below two standard deviations (-2SD) from the median of the reference population are classified as underweight. Finally, the dependent variables for this study are stunting (stunted vs normal), wasting (wasted vs normal) and underweight (underweight vs normal).

#### Independent variables

Birth weight is considered to be the main independent variable or risk factor of interest because the focus of this study is to assess the association between LBW and malnutrition. The BDHS including all DHS in developing countries retrospectively collect information on baby’s size at birth based on mother’s perception as proxy of birth weight by asking question “was the newborn very large, larger than average, average, smaller than average or very small?” This is because majority of births in Bangladesh, like other developing countries, occur at home without proper measurement of birth weight. Actual weight at birth was possble to obtain only for 38% births in 2011 survey. However, the birth weight was reported more often by mothers with higher education and better socio-economic condition and who delivered birth at health facility. Therefore, including birth weight particularly low birth weight in the analysis would introduce selection bias due to such socio-economic differences.

Some recent studies [33–35] conducted using DHS data in developing countries including Bangladesh have shown that about 75% mothers are able to correctly report size at birth, which is about 90% for those births who had LBW (based on reported birth weight), and therefore that mother’s recall on baby’s size could be used as proxy to birth weight. Following these studies we use mather’s recall of baby’s size at birth as proxy to birth weight and created binary exposure with categories ‘LBW’ (very small or smaller than average) and ‘normal birth weight’ (average, larger than average, very large).

It is a well known fact that child malnutrition is the outcome of multiple factors. Hence, in addition to LBW, several other independent variables have been considered following relevant literature [18,19,27]. These include child’s age (categorized when required as <12 months, 12–23 months, 24–59 months following literature), child’s sex (male, female), mother’s education (none, primary, secondary, higher), length of preceding birth interval i.e. time-duration of the current birth from immediate previous birth (first birth, short ‘for interval 9–24 months’, medium ‘for interval 25–48 months’, long ‘for interval 49 months and above’), mother’s height (categorized when required as < = 145cm, >145cm at the avearge height for Bangladeshi women), household socio-economic status (poorest, poorer, rich, richer, richest), area of residence (urban ‘all city corporations and thana head-quarters’, rural ‘all remote areas’), and administrative region (Barishal, Chittagong, Dhaka, Khulna, Rajshahi, Rangpur, Sylhet). The variable household socio-economic status was created by making five equal groups (5 wealth quintiles) based on wealth index calculated from the assets owned by the household using principal component analysis. Details on calculation of the wealth index from household assests can be found elsewhere [15].

### Statistical analysis

To explore the relationship between LBW and malnutrition, risk ratio (RR) of malnutrition comparing children having LBW with those having normal birth weights was calculated from 2x2 table consisting of one of the malnutrition indicators in the column and birth weight status in the row. However, the true relationship between malnutrition and LBW may be distorted by other risk factors associated with both LBW and malnutrition (confounding). To mitigate the influence of a given risk factor or confounder, the association between LBW and malnutrition was assessed separately at each level of that confounder (via stratified analysis). This provides RRs with 95% confidence interval for each level of the confounder. The adjusted RR was then calculated as the weighted average of stratum-specific risk ratios (Cochran-Mantel-Haenszel approach) controlling for the confounder. In the next step significance of interactions was assessed by fitting logistic regression models with log link function containing an interaction term for LBW and the confounder and their corresponding main effects. Separate models were estimated for each of the dependent variables stunting, wasting and underweight that collectively reflect malnutritional status. Finally, the relationship between LBW and child malnutrition was assessed in a multivariate setting by controlling for all possible confounders and significant interactions in a multivariable regression model.

The risk factors ‘child age’ and ‘mother’s height’ were considered as continuous variables in the multivariable model to allow for their full variation. However, these variables were treated as categorical in 2x2 table analysis to make them interpretable. The linearity of the continuous risk factors was examined by including their quadratic terms in the model and found linear relationship with the risk of malnutrition.

As the dataset used in the study is extracted from a multistage cluster survey, all the statistical analyses were conducted allowing for the design effect of complex survey to provide precise confidence interval for RR. Analyses were conducted using a combination of packages “svy”, “epitab” and “binreg” in Stata version 12.

## Results

Table 1 presents the distribution of children with age under five by background characteristics considered in this study. The prevalences of stunting, wasting and underweight in a total of 7530 children are found to be 41%, 16%, and 36%, respectively. The prevalences of LBW and all other background characteristics are very similar to those given in the BDHS report[15].

**Table 1:** Distribution of children under five years by background characteristics.

Variable	Total number of children (N)	Percent
**Stunting**		
Stunted	3063	41.1
Normal	4467	58.9
**Wasting**		
Wasted	1186	15.8
Normal	6344	84.1
**Underweight**		
Underweight	2702	36.2
Normal	4828	63.7
**Birth weight**		
LBW	1259	16.7
Normal	6271	83.2
**Child’s age**		
<12 months	1455	19.3
12–23 months	1422	18.8
24–59 months	4653	61.8
**Child’s sex**		
Male	3846	51.1
Female	3684	48.9
**Mother’s education**		
None	1429	18.9
Primary	2297	30.5
Secondary	3218	42.7
Higher	586	7.8
**Preceding birth interval**		
First birth	2668	35.43
Short	692	9.2
Medium	1815	24.1
Long	2355	31.3
**Mother’s height**		
< = 145 cm	986	13.2
>145 cm	6501	86.8
**Access to Food**		
Limited	1413	18.5
Adequate	2331	81.5
**Socio-economic status**		
Poorest	1664	22.1
Poorer	1462	19.4
Rich	1440	19.1
Richer	1464	19.4
Richest	1500	19.9
**Area of residence**		
Urban	2302	30.6
Rural	5228	69.4
**Administrative region**		
Barishal	821	10.9
Chittagong	1490	19.8
Dhaka	1251	16.6
Khulna	883	11.7
Rajshahi	896	11.9
Rangpur	982	13.1
Sylhet	1207	16.1
**Total**	**7530**	**100.0**

It is evident from data that a higher percentage of children with LBW are malnourished compared to those with normal birth weights. For example, among the children with LBW, 50.9% are stunted, 24.6% are wasted, and 52.1% are underweight compared to 38.6%, 13.9% and 32.6%, respectively in the case of children with normal birth weights (results not shown). These prevalences are still markedly high among children with LBW compared to those with normal birth weights at each level of the confounder in stratified analysis (Tables 2–4). When the strength of associations is quantified using the RR, strong association is found between LBW each of the malnutrition indicators-stunting, wasting, and underweight at each level of the respective confounder (see RRs in Tables 2–4). Even after adjusting for confounders, LBW is found to be significantly associated with these indicators. For example, after controlling for child’s age, the adjusted RR for stunting, wasting and underweight are 1.33 (95% CI: 1.25–1.41), 1.75 (95% CI: 1.56–1.96) and 1.61 (95% CI: 1.51–1.72), respectively. These results suggest that LBW is associated with malnutrition throughout early childhood, i.e, from infancy to five years of age. Further, the interaction effect is found to be statistically insignificant in the logistic regression model containing main and interaction effects of LBW and child’s age (result not shown). This suggests that child’s age does not modify the association between LBW and malnutrition.

**Table 2:** Risk ratios and 95% confidence intervals measuring association between low birth weight (LBW) and stunting.

Confounder categories	Birth weight	Number at risk	% Stunting	RR (95% CI)*	RR (95% CI)**
**Child’s age**					
<12 months	LBW	256	31.8	1.75 (1.41–2.18)	
	Normal	1197	18.1		
12–24 months	LBW	256	61.1	1.31 (1.17–1.47)	1.33 (1.25–1.41)
	Normal	1157	46.5		
25–59 months	LBW	736	53.9	1.26 (1.17–1.36)	
	Normal	3917	42.7		
**Child’s sex**					
Male	LBW	585	50.7	1.32 (1.21–1.45)	
	Normal	3261	38.4		1.32 (1.23–1.40)
Female	LBW	674	51.1	1.31 (1.20–1.43)	
	Normal	3010	38.9		
**Mother’s Education**					
None	LBW	274	59.1	1.16 (1.03–1.30)	
	Normal	1155	51.0		
Primary	LBW	403	57.1	1.29 (1.17–1.42)	
	Normal	1894	44.3		1.28 (1.20–1.36)
Secondary	LBW	515	44.5	1.33 (1.19–1.49)	
	Normal	2703	33.2		
Higher	LBW	67	29.8	1.60 (1.06–2.40)	
	Normal	519	18.7		
**Preceding birth interval**					
First birth	LBW	480	46.9	1.33 (1.12–1.49)	
	Normal	2188	35.1		
Short	LBW	122	60.7	1.21 (1.03–1.43)	
	Normal	570	49.8		1.32 (1.24–1.40)
Medium	LBW	288	57.6	1.28 (1.15–1.43)	
	Normal	1527	44.8		
Long	LBW	369	47.7	1.38 (1.22–1.56)	
	Normal	1986	34.5		
**Mother’s height**					
< = 145 cm	LBW	194	67.1	1.13 (1.01–1.27)	
	Normal	792	59.1		1.28 (1.21–1.38)
>145 cm	LBW	1061	47.9	1.34 (1.25–1.44)	
	Normal	5440	35.7		
**Socio-economic status**					
Poorest	LBW	320	63.2	1.18 (1.08–1.30)	
	Normal	1344	53.2		
Poorer	LBW	262	54.9	1.21 (1.07–1.38)	
	Normal	1200	45.3		
Moddle	LBW	238	49.6	1.28 (1.11–1.48)	1.27 (1.20–1.35)
	Normal	1202	38.8		
Richer	LBW	227	47.6	1.45 (1.24–1.70)	
	Normal	1237	32.9		
Richest	LBW	212	32.5	1.41 (1.13–1.76)	
	Normal	1500	22.8		
**Access to food**					
Limited	LBW	300	62.5	1.23 (1.10–1.22)	
	Normal	1113	50.5		1.30 (1.22–1.37)
Adequate	LBW	996	48.0	1.32 (1.23–1.42)	
	Normal	1897	36.2		
**Area of residence**					
Urban	LBW	362	46.9	1.41 (1.25–1.61)	
	Normal	1940	32.8		1.31(1.23–1.40)
Rural	LBW	897	52.5	1.27 (1.18–1.40)	
	Normal	4331	41.2		
**Administrative region**					
Barishal	LBW	112	49.1	1.19 (0.97–1.47)	
	Normal	709	41.1		
Chittagong	LBW	291	48.8	1.25 (1.09–1.44)	
	Normal	1199	38.7		
Dhaka	LBW	198	53.5	1.30 (1.12–1.50)	
	Normal	1053	41.1		
Khulna	LBW	137	48.2	1.56 (1.27–1.92)	1.31(1.22–1.40)
	Normal	746	30.4		
Rajshahi	LBW	140	45.0	1.50 (1.21–1.86)	
	Normal	756	29.9		
Rangpur	LBW	128	56.3	1.38 (1.16–1.65)	
	Normal	854	40.4		
Sylhet	LBW	253	54.2	1.19 (1.04–1.36)	
	Normal	954	45.5		
**Total**		**7530**	**40.7**	RR (95% CI):1.23 (1.16–1.30)***	

*Risk Ratio (RR) (95% confidence interval (CI)) unadjusted;

** RR (95% CI) adjusted for each confounder;

***RR (95% CI) adjusted for all confounders simultaneously in a multivariable model.

**Table 3:** Risk ratios with 95% confidence intervals measuring association between low birth weight (LBW) and wasting.

Confounder categories	Birth weight	Number at risk	% Wasting	RR (95% CI)*	RR (95% CI)**
**Child’s age**					
<12 months	LBW	256	22.5	1.79 (1.36–2.36)	
	Normal	1197	12.4		
12–24 months	LBW	256	28.7	2.13 (1.68–2.71)	1.75 (1.56–1.96)
	Normal	1157	13.6		
25–59 months	LBW	736	23.9	1.62 (1.39–1.88)	
	Normal	3917	14.6		
**Child’s sex**					
Male	LBW	585	26.2	1.78 (1.52–2.09)	
	Normal	3261	14.5		1.76(1.56–1.97)
Female	LBW	674	23.3	1.73 (1.47–2.04)	
	Normal	3010	13.4		
**Mother’s education**					
None	LBW	274	31.0	2.0 (1.60–2.50)	
	Normal	1155	15.4		
Primary	LBW	403	27.1	1.6 (1.37–2.01)	
	Normal	1894	16.3		1.72(1.53–1.92)
Secondary	LBW	515	21.6	1.71 (1.41–2.08)	
	Normal	2703	12.4		
Higher	LBW	67	7.5	0.70 (0.30–1.68)	
	Normal	519	10.6		
**Preceding birth interval**					
First birth	LBW	480	22.5	1.66 (1.30–2.02)	
	Normal	2188	13.4		
Short	LBW	122	27.1	1.98 (1.38–2.83)	
	Normal	570	13.5		1.75 (1.56–1.97)
Medium	LBW	288	26.1	1.68 (1.33–2.10)	
	Normal	1527	15.5		
Long	LBW	369	25.5	1.87 (1.52–2.30)	
	Normal	1986	13.7		
**Mother’s height**					
< = 145 cm	LBW	194	26.3	1.81 (1.35–2.42)	
	Normal	792	14.5		1.75 (1.56–1.96)
>145 cm	LBW	1061	24.2	1.73 (1.53–1.97)	
	Normal	5440	13.9		
**Socio-economic status**					
Poorest	LBW	320	27.8	1.75 (1.41–2.26)	
	Normal	1344	15.9		
Poorer	LBW	262	21.4	1.33 (1.02–1.74)	
	Normal	1200	15.9		
Moddle	LBW	238	31.5	2.13 (1.68–2.69)	1.72 (1.53–1.93)
	Normal	1202	14.6		
Richer	LBW	227	23.4	1.99 (1.50–2.63)	
	Normal	1237	11.7		
Richest	LBW	212	17.5	1.46 (1.05–2.05)	
	Normal	1500	11.7		
**Access to food**					
Limited	LBW	300	28.9	1.82 (1.45–2.28)	
	Normal	1113	15.8		1.73 (1.54–1.94)
Adequate	LBW	996	23.2	1.70 (1.48–1.95)	
	Normal	1897	13.6		
**Area of residence**					
Urban	LBW	362	23.5	1.85 (1.48–2.30)	
	Normal	1940	12.6		1.74 (1.56–1.96)
Rural	LBW	897	25.1	1.71 (1.49–1.95)	
	Normal	4331	14.5		
**Admisnistrative region**					
Barishal	LBW	112	20.5	1.48 (0.98–2.23)	
	Normal	709	13.7		
Chittagong	LBW	291	25.8	1.92 (1.51–2.44)	
	Normal	1199	13.4		
Dhaka	LBW	198	25.3	1.75 (1.32–2.32)	
	Normal	1053	14.4		
Khulna	LBW	137	22.6	1.75 (1.22–2.52)	1.73 (1.56–1.96)
	Normal	746	12.9		
Rajshahi	LBW	140	31.4	2.25 (1.66–3.05)	
	Normal	756	14.1		
Rangpur	LBW	128	15.6	1.21 (0.78–1.88)	
	Normal	854	12.8		
Sylhet	LBW	253	26.5	1.59 (1.23–2.05)	
	Normal	954	16.3		
**Total**		**7530**	**15.8**	RR (95% CI):1.71 (1.53–1.92)***	

*Risk Ratio (RR) (95% confidence interval (CI)) unadjusted;

** RR (95% CI) adjusted for each confounder;

***RR (95% CI) adjusted for all confounders simultaneously in a multivariable model.

**Table 4:** Risk ratios with 95% confidence intervals measuring association between low birth weight (LBW) and underweight.

Categories	Birth weight	Number at risk	% underweight	RR (95% CI)*	RR (95% CI)**
**Child’s age**					
<12 months	LBW	256	36.1	2.11 (1.72–2.61)	
	Normal	1197	17.0		
12–24 months	LBW	256	56.2	1.83 (1.60–2.11)	1.61 (1.51–1.72)
	Normal	1157	30.5		
25–59 months	LBW	736	56.2	1.47 (1.36–1.58)	
	Normal	3917	38.1		
**Child’s sex**					
Male	LBW	585	52.1	1.67 (1.52–1.83)	
	Normal	3261	31.1		1.58 (1.48–1.68)
Female	LBW	674	52.1	1.51 (1.38–1.65)	
	Normal	3010	34.3		
**Mother’s education**					
None	LBW	274	63.5	1.39 (1.25–1.56)	
	Normal	1155	45.5		
Primary	LBW	403	58.1	1.50 (1.35–1.66)	
	Normal	1894	38.5		1.53 (1.44–1.63)
Secondary	LBW	515	44.1	1.64 (1.46–1.84)	
	Normal	2703	26.6		
Higher	LBW	67	31.5	2.25 (1.48–3.39)	
	Normal	519	13.9		
**Preceding birth interval**					
First birth	LBW	480	46.7	1.59 (1.41–1.78)	
	Normal	2188	29.1		
Short	LBW	122	59.8	1.44 (1.21–1.72)	
	Normal	570	41.6		1.59 (1.49–1.69)
Medium	LBW	288	58.7	1.54 (1.37–1.73)	
	Normal	1527	37.7		
Long	LBW	369	51.5	1.71 (1.52–1.92)	
	Normal	1986	30.5		
**Mother’s height**					
< = 145 cm	LBW	194	64.4	1.39 (1.22–1.59)	
	Normal	792	46.1		1.57 (1.47–1.67)
>145 cm	LBW	1061	49.8	1.62 (1.51–1.74)	
	Normal	5440	30.8		
**Socio-economic status**					
Poorest	LBW	320	65.6	1.39 (1.26–1.53)	
	Normal	1344	47.2		
Poorer	LBW	262	55.7	1.41 (1.24–1.61)	
	Normal	1200	39.3		
Moddle	LBW	238	52.1	1.54 (1.33–1.79)	1.53 (1.44–1.62)
	Normal	1202	33.5		
Richer	LBW	227	45.4	1.87 (1.57–2.22)	
	Normal	1237	24.2		
Richest	LBW	212	34.4	1.82 (1.46–2.28)	
	Normal	1500	18.5		
**Access to food**					
Limited	LBW	300	60.94	1.37 (1.22–1.53)	
	Normal	1113	44.36		1.55 (1.46–1.65)
Adequate	LBW	996	49.27	1.62 (1.51–1.76)	
	Normal	1897	30.23		
**Area of residence**					
Urban	LBW	362	46.7	1.79 (1.56–2.05)	
	Normal	1940	25.9		1.58 (1.48–1.68)
Rural	LBW	897	54.3	1.52 (1.41–1.68)	
	Normal	4331	35.6		
**Administrative region**					
Barishal	LBW	112	49.1	1.35 (1.09–1.67)	
	Normal	709	36.1		
Chittagong	LBW	291	50.5	1.53 (1.33–1.76)	
	Normal	1199	32.8		
Dhaka	LBW	198	56.6	1.78 (1.53–2.07)	
	Normal	1053	31.7		
Khulna	LBW	137	44.5	1.73 (1.38–2.16)	1.58 (1.48–1.68)
	Normal	746	25.7		
Rajshahi	LBW	140	47.9	1.64 (1.33–2.02)	
	Normal	756	29.3		
Rangpur	LBW	128	52.3	1.65 (1.36–2.01)	
	Normal	854	31.3		
Sylhet	LBW	253	58.1	1.44 (1.26–1.64)	
	Normal	954	39.7		
**Total**		**7530**	**35.9**	RR (95% CI):1.47 (1.38–1.56)***	

*Risk Ratio (RR) (95% confidence interval(CI)) unadjusted;

** RR (95% CI) adjusted for each confounder;

***RR (95% CI) adjusted for all confounders simultaneously in a multivariable model.

Similar findings are observed when the association between LBW and malnutrition are adjusted separately for sex of child, mother’s education, length of preceding birth interval, mother’s height, household socio-economic status, area of residence and administrative region. For example, when controlling for mother’s education, the risk of malnutrition is significantly higher among children with LBW than among children with normal birth weights as indicated by the RR values of 1.28 (95% CI: 1.20–1.36), 1.53 (95% CI: 1.44–1.63), and 1.53 (95% CI: 1.44–1.63) for stunting, wasting and underweight, respectively. Similarly, a significantly increased risk of malnutrition (stunting, wasting, underweight) is observed among children with LBW at all levels of the risk factor socio-economic status. The RR is also significantly greater than one indicating that LBW is associated with malnutrition even after taking into account the effect of socio-economic status (Tables 2–4). Interestingly, none of the interactions were statistically significant suggesting that the association between LBW and malnutrition is not modified by maternal education, household economic status or any of the other risk factors.

After adjusting for all confounders simultaneously in a multivariable logistic model, the association between LBW and malnutrition (stunting, wasting, and underweight) remains statistically significant with RR values of 1.23 (95% CI: 1.16–1.30), 1.71 (95% CI: 1.53–1.92) and 1.47 (95% CI: 1.38–1.56) being observed for stunting, wasting and underweight, respectively. Similar findings are observed when the association between LBW and child’s BMI-for-age is investigated (results not shown). Further investigation is also conducted by fitting multivariable linear regression to the continuous Z-score for each of the malnutrition indicators instead of their binary outcome and found similar results (not shown).

## Discussion and Conclusion

This study has examined the association between LBW and malnutrition among children under five years in Bangladesh while adjusting for other known risk factors. In the literature, mother’s education, socio-economic status and length of preceding birth interval are well established risk factors of child malnutrition yet the link between LBW and child malnutrition in Bangladesh has not been thoroughly studied. In this paper, a detailed analysis using retrospective data has been conducted to investigate how and to what extent LBW is associated with the nutritional status of children under five years of age. Such association studies reveal whether there is a systematic variation in the prevalence of LBW among malnouished and healthy children. This in turn may be useful in generating hypotheses and designing future studies that investigate whether LBW has a causal effect on malnutrition.

This study has found a very strong positive association between LBW and malnutrition among children under age five in Bangladesh. For example, the risk of being underweight during the early years of childhood was found to be 47% higher in children with LBW than in children with normal birth weights even after controlling for other factors in a multivariable model. Thus, it appears that babies who are underweight at birth have a tendency to remain underweight during their early childhood. The observed association between birth weight and malnutrition is consistent with the findings of other studies [16,17,26,36–40]. For example, Arifeen et al. [29] described a study of infant growth patterns and their relations to birth weight in low socio-economic conditions in Dhaka, Bangladesh and found that birth weight was the most important determinant of subsequent growth status during infancy in this population. A number of studies have reported that poor birth weight leads to increased risk of disease morbidity and mortality due to malnutrition [33,38,41].

On the other hand, the link between LBW and child malnutrition could possibly be described by the increased vulnerability of children with LBW to infections, such as, diarrheal and lower respiratory infections and the increased risk of complications including sleep apnea, jaundice, anemia, chronic lung disorders, fatigue and loss of appetite compared to children with normal birth weights [34,42–44]. Greater morbidity among children with LBW results in poor physical growth and development that is perceived as malnutrition. A similar explanation has been given by Ramakrishnan [45] based on a study on infant girls born with LBW in developing countries. He found that children with LBW experienced growth failure during early childhood and into the adolescence period and the ensuing malnutrition ultimately led to increased risk of maternal complications in later life.

However, perhaps the most interesting finding of this study is that the well known risk factors for child malnutrition, such as, mother’s education, length of preceding birth interval and socio-economic conditiondid notmodify the association between LBW andmalnutrition. The implications of this finding are important. This suggests that once a baby is born underweight, the risk of becoming malnourished during the first five years of life is higher compared to a baby of normal birth weight even if the mother is educated, household socio-economic conditions are good, and the preceding birth interval is long. This may be one explanation as to why prevalence of malnutrition has remained high in Bangladesh during the last decade despite marked improvements with respect to each of these factors over the same period.

Based on the results of the current investigation, it is our opinion that a reasonable prescription for addressing the child malnutrition problem in Bangladesh would be to reduce the prevalence of LBW in addition to spacing births and improving mother’s education and socio-economic well-being. Since child malnutrition has its origins in the foetal period [40], favorable socio-demographic conditions duirng the postnatal period cannot fully compensate for the intial setback. According to the national Low Birth Weight Survey 2004 [28], the prevalence of LBW (weight below 2500 gm) is 35.6%, which is still very high compared to developed countries. Therefore, special attention is required for reducing prevalence of LBW.

As discussed in the medical literature, the main reason for LBW in developing countries is intra-uterine growth retardation (IUGR) [28,30]. A baby who suffers from IUGR as a foetus is effectively born malnourished. About half of all IUGR cases in developing countries are attributable to mother’s malnutrition, low maternal weight and stature at conception and low weight gain during pregnancy [28,30]. Iron deficiency and anemia are also associated with IUGR and hence with LBW [46,47]. In addition, very young mothers (age <20) are more likely to have babies suffering from IUGR and are therefore at a greater risk of giving birth to LBW infants [38,48]. This is because a young mother demands double set of nutrition as she struggles to complete her own growth. Married adolescent girls must be counseled by their parents or healthcare providers about the importance of not getting pregnant until they complete their own growth. All these issues suggest that special emphasis should be given to awareness building programs targeting the population of adolescent girls and pre-pregnant women so that all women enter pregancy with optimal health and nutrition. Most importantly, expecting mothers require access to quality antenatal health care services.

Unfortunately, the percentage of women in Bangladesh seeking health care during pregnancy from trained health care providers is still below 50% [15], which is is substantially lower (37%) in rural areas where the majority of the population lives. Apart from lack of awareness, these dismal figures are due to the unavailability of quality health care providers particularly in rural areas. According to the Directorate General of Health Science (DGHS) [49], 28% posts for MBBS doctors and 20% posts for nurses in upazila based goverment hospitals are still vacant. A large number of trained health care providers are available in urban-based private clinics, but the low income families cannot afford the service. On the other hand, government hospitals and clinics being subsidized are affordable and thus the goverment should take necessary steps to fill the vacant posts by recruiting trained health care providers.

In conclusion, this study has provided evidence of a strong link between LBW and child malnutrition. This link is crucial to the formulation of successful interventions aimed at reducing the prevalence of child malnutrition. For a quick improvement in nutritional status of children under five in Bangladesh, it is very urgent to undertake targeted interventions aimed at reducing prevalence of children with LBW in addition to improving women’s education and other socio-demographic conditions.

### Limitation of the study

One caveat regarding measurement of the main exposure variable LBW should be mentioned. Since the BDHS 2011 collected information retrospectively, actual birth weight measurements were unavailable, so that LBW was defined based on mother’s perception of the size of child at birth. Underreporting is therefore expected since most mothers would be able to recall whether the baby was underweight only if the baby was very small in size (i.e. << 2500gm). Thus, the prevalence of LBW was found to be 16.7% in our study, which is much lower than 35.6% obtained by the 2004 National Low Birth Weight Survey [28] that measured LBW from actual birth weights. The prevalence of LBW obtained in this study is consistent with other studies [33,34] that measure poor size at birth as a proxy for LBW based on actual birth weights. Our estimate is however conservative as there is high chance that very low birth weight babies (pre-term or full term) will be correctly reported and very low chance, if any, that babies of normal birth weight will be misclassified as LBW using mother’s recall method.
